# Generating the Visual Biofeedback Signals Applicable to Reduction of Wrist Spasticity: A Pilot Study on Stroke Patients

**DOI:** 10.29252/nirp.bcn.9.1.15

**Published:** 2018

**Authors:** Afsane Zadnia, Hamid Reza Kobravi, Mania Sheikh, Hossein Asghar Hosseini

**Affiliations:** 1. Research Center of Biomedical Engineering, Mashhad Branch, Islamic Azad University, Mashhad, Iran.; 2. Department of Physiotherapy, School of Paramedical Sciences, Mashhad University of Medical Sciences, Mashhad, Iran.

**Keywords:** Biofeedback, Muscle spasticity, Stroke, Electromyography

## Abstract

**Introduction:**

Application of biofeedback techniques in rehabilitation has turned into an exciting research area during the recent decade. Providing an appropriate visual or auditory biofeedback signal is the most critical requirement of a biofeedback technique. In this regard, changes in Surface Electromyography (SEMG) signals during wrist movement can be used to generate an indictable visual biofeedback signal for wrist movement rehabilitation via SEMG biofeedback. This paper proposes a novel methodology for selecting the most appropriate features out of wrist muscle SEMG signals.

**Methods:**

To this end, the surface EMG signals from flexor and extensor muscle groups during wrist joint movements were recorded and analyzed. Some linear and nonlinear features in frequency, time, and time-frequency domains were extracted from the recorded surface EMG signals of the flexor and extensor muscles. Experiments and analyses were performed on ten healthy subjects and four stroke patients with wrist muscle spasticity as the movement disorder subjects. Some heuristic feature selection measures were applied. The main motivation behind choosing applied heuristic feature selection measures was meeting. In the first step, the designed visual biofeedback signal should indicate a healthy wrist motion profile as its successful tracking by the patient guarantees rehabilitation. In addition, the visual biofeedback signal should be a smooth curve thus preventing the patient from discomfort while tracking it on a monitor during the biofeedback therapy.

**Results:**

In this pilot study, after using the introduced feature selection measures, quantitative and qualitative analyses of the extracted features indicated that Shannon entropy is the most appropriate feature for generating a visual biofeedback signal as a healthy wrist motion profile to improve the ability of stroke patients in controlling wrist joint motion. In addition, it was shown that when the wrist joint moves between a flexed and rest position, the flexor muscle EMG signal should be used for generating a visual biofeedback signal. However when the wrist joint moves between a rest position and an extended position, the extensor muscle EMG signal is appropriate for providing a visual biofeedback signal. It is worth noting that the achieved pilot study results should be confirmed by the future studies with larger samples.

**Conclusion:**

According to the obtained results, it can be concluded that among the analyzed features, the Shannon entropy was the most appropriate feature. It can be employed for generating a visual biofeedback signal for reduction of spasticity in patients with stroke.

## 1. Introduction

One of the consequences of stroke is the increase in muscle tone due to the hyper-excitability of the stretch reflex (Doğan-Aslan, Nakipoğlu-Yüzer, Doğan, Karabay, & Özgirgin, 2012; Alibiglou, Rymer, Harvey, & Mirbagheri, 2008). Spasticity can result in a loss of normal motor patterns, reduced flexibility, impaired posture, decreased functional mobility, and joint pain (Doğan-Aslan et al., 2012; Alibiglou et al., 2008; Armagan, Tascioglu, & Oner, 2003). Therefore, spasticity reduction, especially in the upper limbs, significantly affects the quality of life of post-stroke patients. Prevailing approaches for upper extremity rehabilitation, such as conventional exercise programs, proprioceptive neuromuscular facilitation techniques, muscle strengthening and physical conditioning programs, neurophysiologic approaches, and functional electrical stimulation (Armagan et al., 2003) do not always succeed in restoring upper limb function or may require a significant number of treatment sessions.

One of the most effective as well as noninvasive upper extremity rehabilitation approaches is biofeedback (Doğan-Aslan et al., 2012; Armagan et al., 2003; Huang, Wolf, & He, 2006; Wolf, 1983; Merletti & Parker, 2004). Biofeedback enables patients to learn how to change their physiological activities to improve their performance (Huang et al., 2006; Nelson, 2007). In such an approach, physiological activities, such as brainwaves, heart function, breathing, muscle activity, skin temperature, and joint angle are recorded and then feedback signals are generated according to them. The feedback signals are displayed through a space-based virtual reality, an auditory pitch or volume, or a mechanical tactile system (Huang et al., 2006). The choice of a biofeedback vehicle depends on the motor control mechanism, training task, and therapeutic goals (Huang et al., 2006).

Among the biomedical signals employed for biofeedback techniques, EMG signals are extensively used in the rehabilitation field as they provide significant information about the activation pattern of muscles (Doğan-Aslan et al., 2012; Armagan et al., 2003; Huang et al., 2006; Wolf, 1983; Merletti & Parker, 2004). Biofeedback training based on EMG signals improves muscle control ability of joint motion (Armagan et al., 2003; Huang et al., 2006). In a study, the effect of EMG biofeedback treatment on wrist flexor muscle spasticity and upper extremity motor function has been evaluated (Doğan-Aslan et al., 2012).

This research has found the positive effect of EMG biofeedback on hemiplegia rehabilitation (Doğan-Aslan et al., 2012). Armagan et al. have also observed the efficacy of EMG biofeedback treatment on the functional recovery of a hemiplegic hand (Armagan et al., 2003). Findings of another study verified the effect of EMG biofeedback, along with conventional physiotherapy, on improving hand function in subacute stroke subjects (Harishchandre & Singaravelan, 2012). Feature selection is the critical issue in implementing EMG biofeedback systems for the generation of effective biofeedback to the patients. Since the muscular characteristics of individual patients are completely different, EMG amplitude thresholds cannot be considered as appropriate features. Many researchers, however, have demonstrated the nonlinear process of muscle contraction and EMG signal behavior (Arjunan & Kumar, 2007; Gupta, Suryanarayanan. & Reddy, 1997; Hu, Wang, & Ren, 2005; Acharya, Ng, Swapna and Michelle, 2011; Padmanabhan & Puthusserypady, 2004; Lei, Wang, & Feng, 2001; Hassan, Terrien, Marque and Karlsson, 2011). Through a heuristic systematic approach, the current study selected the most appropriate nonlinear feature from wrist muscle EMG signals and the most suitable muscles for the effective generation of a visual biofeedback signal for wrist movement rehabilitation via Surface Electromyography (SEMG) biofeedback.

## 2. Methods

### 2.1. Participants

Ten healthy people and four stroke patients volunteered to participate in this study. Table 1 presents the demographic and clinical features of participants aged 40–70 years old without any systemic disease as well as perceptual disorder. The stroke patients were hemiplegic and suffered from wrist muscle spasticity. Modified Ashworth Scale (MAS) scores of patients were assessed by a physiotherapist (Bohannon & Smith, 1987). Also a consent form was signed by all participants.

**Table 1.** Demographic and clinical features of stroke and healthy groups, data are presented as Mean±SD or proportion

**Features**	**Patient (n=4)**	**Healthy (n=10)**	**ANOVA Test**
Age, y	61±13.44	53.8±9.11	P>0.05
BMI^a^, kg/m^2^	29.4±5.12	29.04±5.12	P>0.05
Sex, female/male	1 (F) /3 (M)	1 (F) /9 (M)	P>0.05
Duration after stroke, mon	4.82±4.29	-	
Stroke type, Isc^b^/Hem^c^	2 (F) /2 (M)	-	
Side of hemiparesis, R^d^/L^e^	3 (F) /1 (M)	-	
MAS^f^ measure	1.1+0.2	-	

a BMI: Body Mass Index;

b Isc: Ischemic;

c Hem: Hemorrhage;

d R: Right;

e L: Left;

f MAS: Modified Ashworth Scale

### 2.2. Experimental protocol and preprocessing

**Figure 1.**
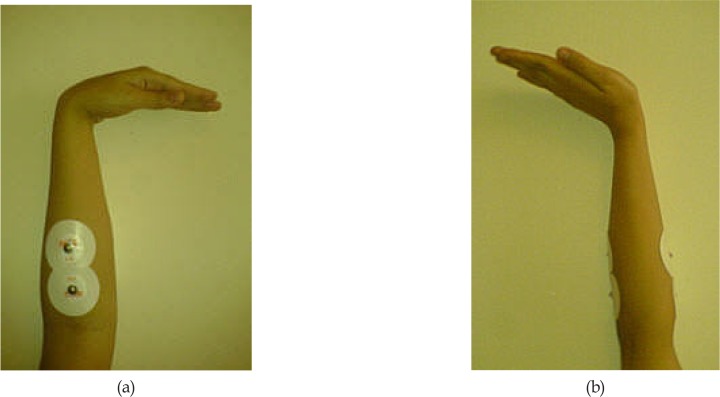
The subjects moved their wrist joint between (a) a flexed position and (b) an extended position

**Figure 2.**
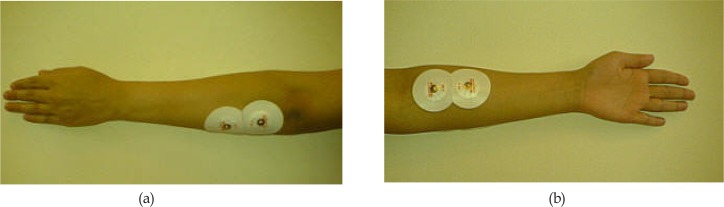
Electrode placement on the (a) wrist extensor and (b) flexor muscle groups

Subject was seated in a quiet room in a comfortable position next to the device with the wrist placed on a pillow at full flexion. Then, the subject was asked to move the wrist joint from a fully flexed position to fully extended position (Figure 1). The experiment lasted between 3 to 22 seconds depending on the subject’s performance. Each subject performed 10 trials. During the movement, EMG signals of wrist extensor and flexor muscles were recorded by a PowerLab/4SP system (ADInstruments Pty Ltd., Australia). Circular Ag/AgCl surface electrodes were placed in the direction of muscle fibers for recordings (Merletti & Parker, 2004). Figure 2 shows the placement of electrodes. For holding down the impedance, the skin was cleaned with 70% alcohol before the recordings. The EMG signals were sampled with a frequency of 2 kHz and filtered through a bandpass filter (10–500 Hz) and a notch filter (50 Hz). The signal was then normalized by the Maximum Voluntary Contraction (MVC) of the extensor and flexor muscles.

### 2.3. Feature extraction

The recorded and preprocessed EMG signals were divided into 1-s time windows during which some linear and nonlinear features were extracted in an off-line fashion. The time course of changes in features was monitored, too. Application of biofeedback system in real-time conditions requires low computational cost of features. In addition, very short data windows (1 second) need to be created. Thus, some specific features were analyzed to guarantee these conditions. Linear features include Root Mean Square (RMS), average linear envelopes, peak, Zero Crossing Rate (ZCR), Median Frequency (MDF), Mean Frequency (MNF), and wavelet coefficient. Shannon entropy was calculated as a nonlinear feature. Analyzed features are described in the following sections.

#### 2.3.1. Root mean square and peak

We used RMS value to quantify the SEMG signal as it reflects the physiological activity in the motor unit during contraction (Fukuda et al., 2010). The RMS of the SEMG signal is indicative of the firing frequency, duration, and velocity of the myoelectric signal. The increment of this feature marks the recruitment of extra motor units to produce constant force.

(1) $$\mathrm{RMS}=\sqrt{\frac{1}{N}\sum_{i=1}^{N}x_{i}^{2}}$$,

where x_i_ is the i^th^ sample of a signal and N is the number of samples in each frame (Cifrek, Medved, Tonković, & Ostojić, 2009). Also, the maximum amplitude of EMG signals during each 1-s time window was calculated.

#### 2.3.2. Average linear envelopes

The linear envelope of EMG signals can be calculated by filtering the full-wave rectified signal through a low pass filter - a butterworth filter of order 6 and cut off frequency of 10 Hz - followed by linear averaging during each 1-s time window (Merletti & Parker, 2004).

#### 2.3.3. Zero crossing rate

This rate corresponds to the number of times that a wave form passes through zero during each 1-s time window. The zero crossing rate of the EMG signal can give a measure of the motor-unit activity in a muscle which is independent of the position of electrodes relative to the motor-unit being picked up. This measure can be calculated as follows.

(2) $$\mathrm{ZCR}=2\left[\frac{\int_{0}^{\frac{f_{s}}{2}}f^{2}X(f)df}{\int_{0}^{\frac{f_{s}}{2}}X(f)df}\right]$$,

where X(f) is the Power Spectral Density (PSD) of a signal, x(t), and f_s_ is the sampling frequency (Cifrek, Medved, Tonković, & Ostojić, 2009).

#### 2.3.4. Median frequency and mean frequency

Mean Frequency (MNF) and Median Frequency (MDF), two frequency indices, are computed using Equation 3 and Equation 4 .

(3) $$\mathrm{MNF}=\frac{\int_{0}^{\infty }\omega P(\omega )d\omega }{\int_{0}^{\infty }P(\omega )d\omega }$$

(4) $$\int_{0}^{MDF}P(\omega )d\omega =\int_{MDF}^{\infty }P(\omega )d\omega =\frac{1}{2}\int_{0}^{\infty }P(\omega )d\omega$$,

where P(ω) is the PSD of the EMG signal and ω is the frequency variable (Georgakis, Stergioulas and Giakas, 2003). In this study, these features were calculated during each 1-s time window.

#### 2.3.5. Wavelet analysis

During each 1-s time window, the EMG signals were decomposed into four levels by the seventh order of Daubechies wavelets and the coefficients of the fourth level were employed for analyses (Phinyomark, Limsakul, & Phukpattaranont, 2011).

#### 2.3.6. Shannon entropy

Shannon entropy, as a nonlinear feature, is a simple quantitative measure of uncertainty and complexity in a data set (Phung, Tran, Ma, Nguyen, & Pham, 2014). Neurological disorder affecting muscle activation pattern can change the complexity and entropy of EMG signal. In the current study, the Shannon entropy of the EMG signal was computed during each 1-s time window. The Shannon entropy can be computed for a particular experimental condition with a set of M possible outcomes as follows.

(5) $$\mathrm{ShEn}=-\sum_{j=1}^{M}p_{j}\ln(p_{j})$$,

where p_j_ is the probability of the j^th^ outcome (Kaufman, Zurcher, & Sung, 2007).

### 2.4. Feature selection measures

The main objective of the current study was to choose appropriate features to provide visual biofeedback signals. For this purpose, some heuristic feature selection measures were introduced and employed. Selected features, however, are determined by the proposed selection measures. The preprocessed EMG signals were divided into 1-s time windows, during which some linear and nonlinear features were extracted under off-line conditions. The time course of extracted features were plotted as the change curves, then some indices, such as gap, SDEP, and the power spectral density were analyzed as selection measures for choosing the most appropriate feature of EMG signals and also to find the most suitable muscles for generating a visual biofeedback signal for the wrist movement. Finally, the selected feature is mapped to the appropriate biofeedback signals on the screen perused by patients during biofeedback therapy. As a result, a desired feature has to satisfy the two conditions explained below.

#### 2.4.1. Discrimination condition

**Figure 3.**
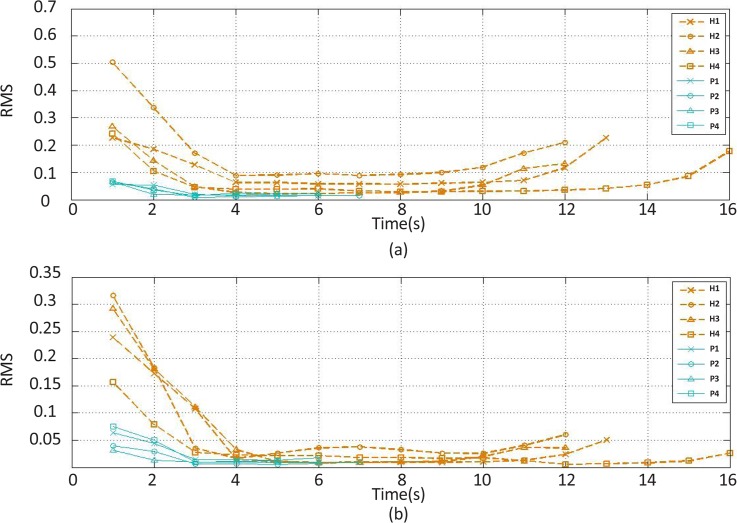
RMS feature changes of the (a) extensor and (b) flexor muscles’ EMG signal over time H stands for healthy subjects and P for stroke patient.

**Figure 4.**
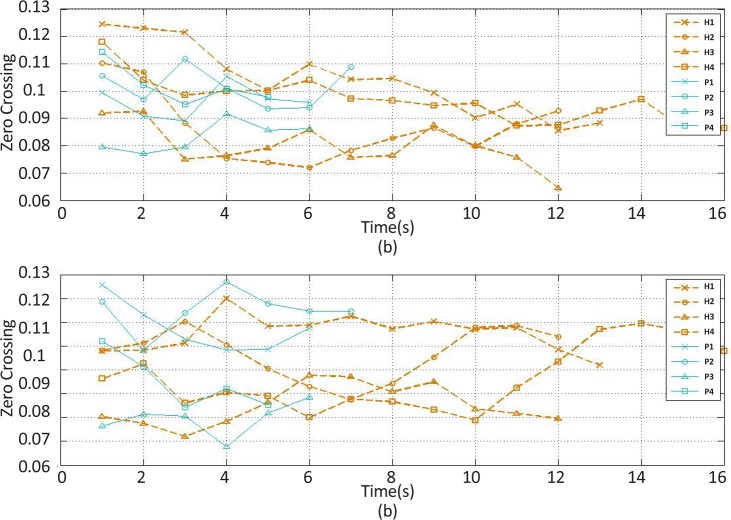
The zero crossing feature changes of the (a) extensor and (b) flexor muscles’ EMG over time H stands for healthy subject and P for stroke patients.

**Figure 5.**
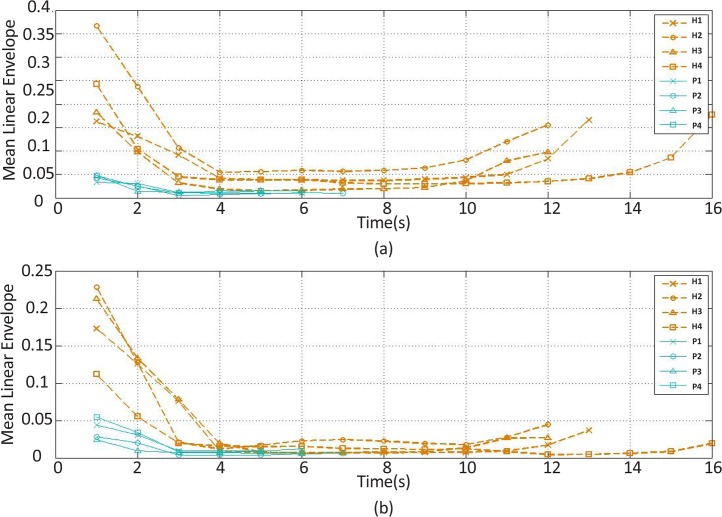
The mean linear envelope feature changes of the (a) extensor and (b) flexor muscles’ EMG signal over time H stands for healthy subject and P stands for stroke patient.

**Figure 6.**
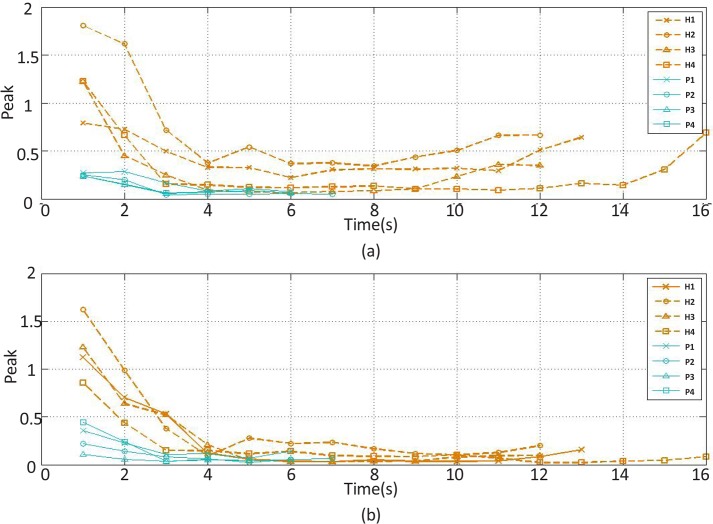
The peak feature changes of the (a) extensor and (b) flexor muscles’ EMG signal over time H stands for healthy subject and P for stroke patient

**Figure 7.**
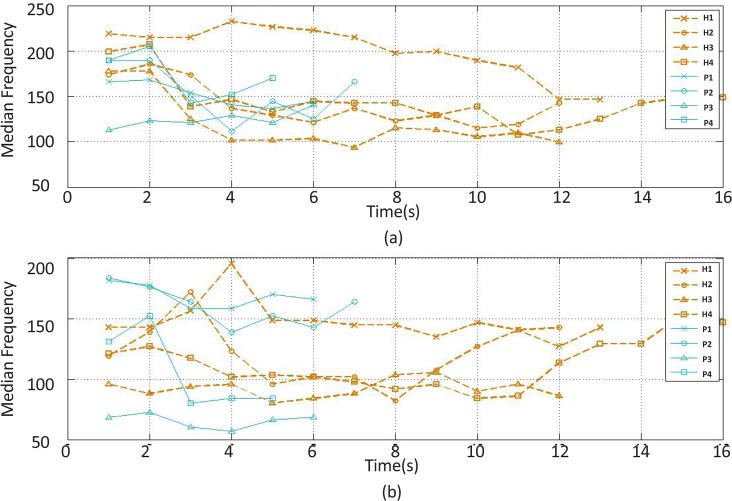
The median frequency feature changes of the (a) extensor and (b) flexor muscles’ EMG signal over time H stands for healthy subject and P for stroke patient.

**Figure 8.**
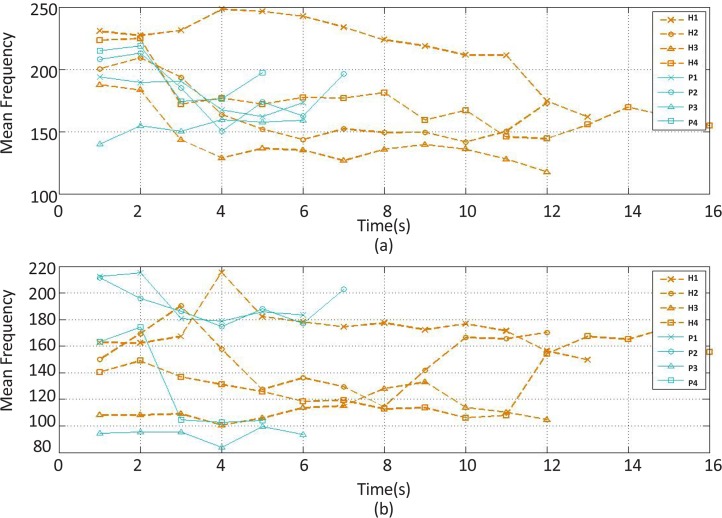
The mean frequency feature changes of the (a) extensor and (b) flexor muscles’ EMG signal over time H stands for healthy subject and P for stroke patient.

**Figure 9.**
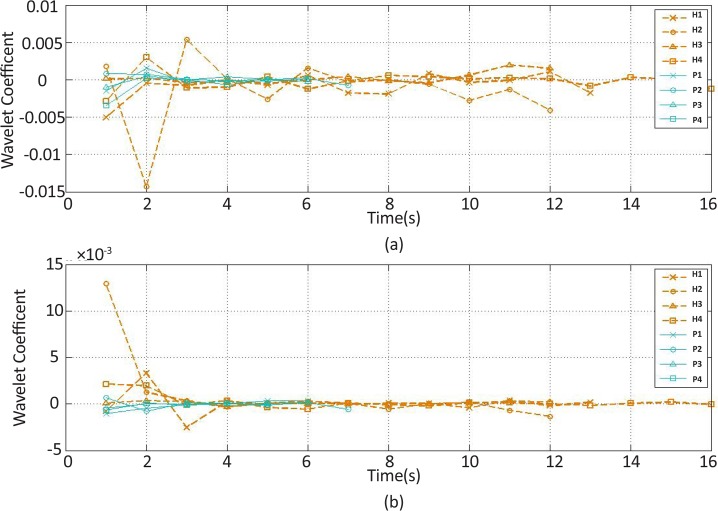
The wavelet coefficient feature changes of the (a) extensor and (b) flexor muscles’ EMG signal over time H stands for healthy subject and P for stroke patient.

**Figure 10.**
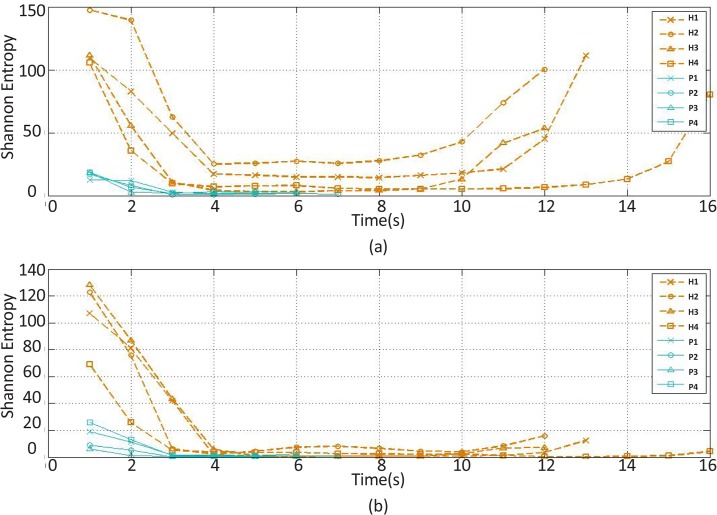
The shannon entropy feature changes of the (a) extensor and (b) flexor muscles’ EMG signal over time H stands for healthy subject and P for stroke patient.

**Figure 11.**
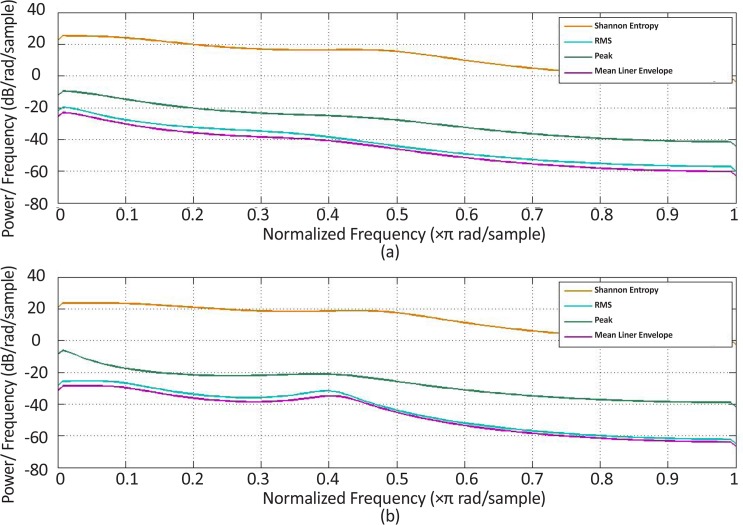
Power spectrum density estimation for one healthy subject (a) Extensor muscle group, (b) Flexor muscle group

**Figure 12.**
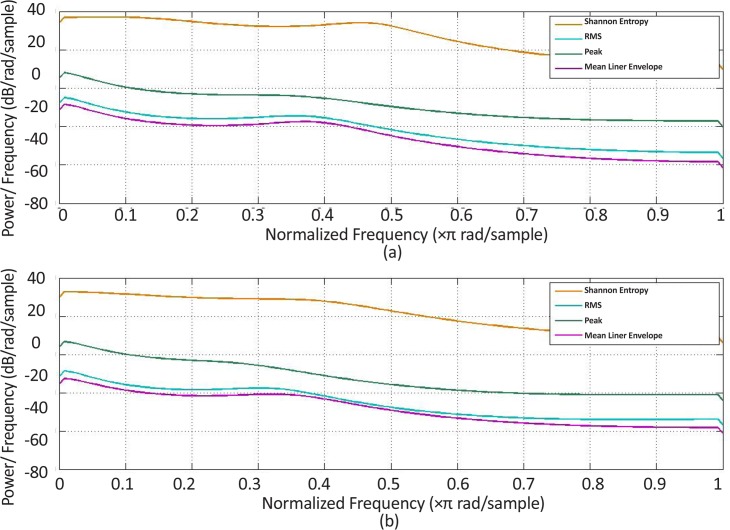
Power spectrum density estimation for one stroke patient subject (a) Extensor muscle group, (b) Flexor muscle group

The selected feature should clearly discern a healthy muscle from the one with spasticity while exhibiting the least deviations among each subject group. Obviously, the change curves of the features extracted from the extensor and flexor muscles had three extreme points (Figure 3-12). The first extreme point was extracted from the first 1-s time window (the wrist joint in the flexed position), the second from the median 1-s time window (the wrist joint in the rest position), and the third from the last 1-s time window (the wrist joint in the extended position). Two heuristic selection measures, namely gap and SDEP were used to select the feature with both the greatest geometric distance between the corresponding change curves of healthy subjects and that of stroke patient subjects with the least standard deviation of extreme points corresponding to the change curves of the healthy subjects.

There is a gap between the change curves illustrating the change in the features of healthy subjects and stroke patients over time. The quantification of this gap was obtained by calculating the difference between the maximum highest points of the change curves of healthy subjects and the maximum highest points of the change curve of the stroke patients. The greater the gap value, the best discrimination ability of the corresponding feature.

As mentioned, the standard deviation of the corresponding extreme points to the selected feature change curves of healthy subjects must be the lowest. Thus, the mean of the extreme points can be considered as three desired motion states achieved during wrist movement in healthy subjects. The Standard Deviation of Extreme Points (SDEP) is computed as a selection measure. Typically, whenever the obtained SDEP has less value, the corresponding feature is more appropriate for generating a visual biofeedback signal indicating desired motion states for the patients.

#### 2.4.2. Smooth changes condition

The visual biofeedback signal is provided to the patient by mapping the selected feature changes to a trajectory or point position changes on a monitor set located in front of the patient. Thus, the smoothness of feature changes during wrist joint movement can be a significant property in that the patient should not feel discomfort or become perplexed. The power spectral density of change curves related to feature changes is a measure showing the smoothness or abruptness of each feature change. The lower energy of higher frequency bands signifies a lower rate of change. The changes in power spectral density over time indicate the fluctuation rate of the extracted feature.

## 3. Results

The recorded and preprocessed EMG signals were divided into some 1-s time windows, and then some linear and nonlinear features were extracted under off-line conditions. The time courses of features were analyzed. Figures 2–10 present the results for four sample healthy subjects along with the results for four stroke patients. Figures 2–10 show feature changes over time. Since the time courses of the features were similar for all healthy subjects, they are demonstrated only for four subjects in each group to see more evident changes. The features were extracted from the EMG signal of extensor and flexor muscles. According to the current study explanations, the feature selection measures were computed to determine the feature that satisfies the desired conditions.

Table 2 shows the mean of the extremes and SDEP based on the data of healthy subjects. According to the results, for the extended position and the baseline position, the SDEP of Shannon entropy extracted from the extensor muscle was the highest among the SDEPs of the other features. Also, for the flexed position, the SDEP of Shannon entropy extracted from flexor muscle was the highest among the SDEPs of the other features.

**Table 2.** Mean of extremes and SDEP based on data of the healthy subjects with the wrist joint in the baseline, flexed, and extended position

**Feature**	**Mean±SD**							

	**Entropy**		**Mean Envelope**		**RMS**		**Peak**	

**Muscle**	**Flexor**	**Extensor**	**Flexor**	**Extensor**	**Flexor**	**Extensor**	**Flexor**	**Extensor**
Baseline position	1.42±2.93	3.1±5.01	0.01±0.01	0.01±0.01	0.01±0.01	0.02±0.02	0.05±0.07	0.09±0.1
Flexed position	82.41±24.03	66.74±39.79	0.14±0.04	0.13±0.09	0.2±0.06	0.18±0.13	1.02±0.35	0.78±0.54
Extended position	8.61±6.51	52.27±28.69	0.02±0.01	0.08±0.1	0.04±0.02	0.13±0.05	0.14±0.08	0.44±0.18

Table 3 shows the mean of the gaps. Results indicate that for each position, the mean of the gap for the Shannon entropy extracted from the extensor muscle is the highest among the means of the gap of the other features.

**Table 3.** Computed gaps with the wrist joint in the baseline, flexed position, and extended position

**Feature**	**Gap**							

	**Entropy**		**Mean Envelope**		**RMS**		**Peak**	

**Muscle**	**Flexor**	**Extensor**	**Flexor**	**Extensor**	**Flexor**	**Extensor**	**Flexor**	**Extensor**
Baseline position	9.85	19.53	0.02	0.04	0.03	0.07	0.19	0.27
Flexed position	34.45	104.86	0.11	0.32	0.14	0.45	0.34	1.88
Extended position	14.22	114.72	0.03	0.56	0.81	−0.2	−0.02	0.79

Figures 11 and 12 present the estimated power spectrum density of the obtained curves in both healthy and stroke subjects, respectively. The results indicate that the power spectral density of the curves indicating changes of the Shannon entropy value over time have the maximum values at low frequencies in comparison with those related to the other features. Such results demonstrate that, among all the other features, the Shannon entropy had experienced the smoothest changes over time.

### 3.1. Generating the visual biofeedback signal

**Figure 13.**
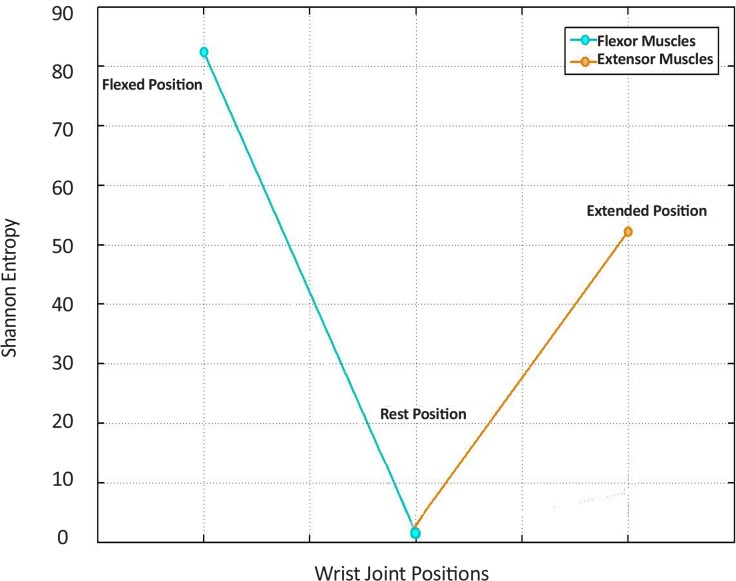
Mean of the computed extreme points of the Shannon entropy change curve for the healthy subjects The left most point, the middle point and the right most points in the Figure are the mean values of the computed Shannon entropy extracted from three time windows of 1-s duration of the data for healthy subjects. Three 1-s time windows are related to the SEMG signals of the wrist flexor muscle (while the joint was at flexed position), the wrist flexor muscle (while the joint was at flexed position), and the wrist extensor muscle (while the joint was at flexed position), respectively. Two straight lines were designed to connect the points to each other, consecutively.

According to results, Shannon entropy adequately satisfies the necessary conditions of feature selection. Therefore, in the next step, a visual biofeedback signal is produced by employing the Shannon entropy feature. In Figure 13, three bold points mark the mean of the computed extreme points of the Shannon entropy change curve for the healthy subjects. The first point extracted from the first 1-s time window (the flexed position) was related to the SEMG signals of wrist flexor muscles.

The second point extracted from the median 1-s time window (the rest position) was related to the SEMG signals of wrist flexor muscles. The third point extracted from the last 1-s time window (the extended position) was related to the SEMG signals of extensor muscles. In fact, these three points should be feedbacked to the patient. Also, the changes of the extracted Shannon entropy over the time should be returned to the patient as the visual biofeedback signal. The patient must try to have the generated visual biofeedback signal reach the set points. When the subject attempts to move his or her wrist joint from a flexed position to an extended position and the trajectory reaches each set point, the wrist joint has positioned itself in the desired position (the proper motion state is reached).

## 4. Discussion

The position of a stroke patient’s wrist joint may be in an unwanted flexed position because of post-stroke spasticity. The SEMG biofeedback system can be effective in the rehabilitation of wrist joint movement. In the current study, a novel approach is proposed for generating SEMG-based visual biofeedback signal for spasticity reduction in stroke patients. In the presented approach, three set points are displayed on the monitor screen. An additional indicator on the screen is shown which implicitly indicates the current position of the wrist joint. The difference between the position of this indicator and the set points is the signal which was feed backed to the patients. Furthermore, the patient must attempt to move his or her wrist joint such that the indicator will move from one set point to another set point consecutively.

Correct tracking of the set points by the patient produces movement of wrist joint from an undesired flexed position to an extended position that can result in spasticity reduction of the flexor muscle of wrist joint. The changes of some extracted features were analyzed and compared in such a manner that the most suitable feature for the design of a set point and indicators could be selected. Thus, the extracted features were compared from three points of views that addressed better discriminative features between the stroke patients and healthy subjects. They are the least computed SDEP of features related to healthy subjects, with the least fluctuation rate in the time course. The extremes of the selected features of healthy subjects can be used to design the set points on the screen monitor, which the stroke patient subjects must try to track consecutively. The values of the selected features computed over time can be used to provide the visual biofeedback signal observed on the monitor screen. It should be emphasized that the computational cost of the extracted features must be low for use in real-time contexts.

According to the obtained results, Shannon entropy was selected as the most appropriate feature among the computed features. Accordingly, Shannon entropy can be employed for generating a visual biofeedback signal for the patients. The Shannon entropy changes that occur over time should be generated as a varying trajectory which moves towards the set points when the patient attempts to move properly his or her wrist joint from the flexed position to extended one. The proposal can be useful for designing a biofeedback rehabilitation system based on visual biofeedback for reducing wrist spasticity after a stroke. Of course, after this stage the experimental studies on patients should be carried out. Since the stroke patient selection and stroke patient instruction are time-consuming processes, the current pilot study decided to separately report its results on SEMG analysis for the generation of an appropriate visual biofeedback signal. These results should be confirmed using the analyses of more recorded data from large size samples in future.

## References

[B1] Acharya U. R. Ng E. Y. K. Swapna G. Michelle Y. S. L. (2011). Classification of normal, neuropathic, and myopathic electromyograph signals using nonlinear dynamics method. Journal of Medical Imaging and Health Informatics, 1(4), 375–80. doi: 10.1166/jmihi.2011.1054

[B2] Alibiglou L. Rymer W. Z. Harvey R. L. Mirbagheri M. M (2008). The relation between Ashworth scores and neuromechanical measurements of spasticity following stroke. Journal of Neuro-Engineering and Rehabilitation, 5(1), 18. doi: 10.1186/1743-0003-5-18 PMC2515334 18627628

[B3] Arjunan S. P. Kumar D. K. (2007). Fractal theory based Non-linear analysis of sEMG. Paper presented at the 3rd International Conference on Intelligent Sensors, Sensor Networks and Information Melbourne, Australia, 3–6 December 2007. doi: 10.1109/issnip.2007.4496901

[B4] Armagan O. Tascioglu F. Oner C (2003). Electromyographic biofeedback in the treatment of the hemiplegic hand. American Journal of Physical Medicine & Rehabilitation, 82(11), 856–61. doi: 10.1097/01.phm.0000091984.72486.e0 14566153

[B5] Bohannon R. W. Smith M. B (1987). Interrater reliability of a modified ashworth scale of muscle spasticity. Physical Therapy, 67(2), 206–7. doi: 10.1093/ptj/67.2.206 3809245

[B6] Cifrek M. Medved V. Tonković S. Ostojić S (2009). Surface EMG based muscle fatigue evaluation in biomechanics. Clinical Biomechanics, 24(4), 327–40. doi: 10.1016/j.clinbiomech.2009.01.010 19285766

[B7] Doğan Aslan M. Nakipoğlu Yüzer G. F. Doğan A. Karabay İ. Özgirgin N (2012). The effect of electromyographic biofeedback treatment in improving upper extremity functioning of patients with hemiplegic stroke. Journal of Stroke and Cerebrovascular Diseases, 21(3), 187–92. doi: 10.1016/j.jstrokecerebrovasdis.2010.06.006 20880720

[B8] Fukuda T. Y. Echeimberg J. O. Pompeu J. E. Lucareli P. R. G. Garbelotti S. Gimenes R. O. (2010). Root mean square value of the electromyographic signal in the isometric torque of the quadriceps, hamstrings and rachial biceps muscles in female subjects. The Journal of Applied Research, 10(1), 32–9.

[B9] Georgakis A. Stergioulas L. K. Giakas G (2003). Fatigue analysis of the surface EMG signal in isometric constant force contractions using the averaged instantaneous frequency. IEEE Transactions on Biomedical Engineering, 50(2), 262–5. doi: 10.1109/tbme.2002.807641 12665043

[B10] Gupta V. Suryanarayanan S. Reddy N. P (1997). Fractal analysis of surface EMG signals from the biceps. International Journal of Medical Informatics, 45(3), 185–92. doi: 10.1016/s1386-5056(97)00029-4 9291030

[B11] Harishchandre M. S. Singaravelan R. M (2012). Effectiveness of EMG biofeedback on improving hand function in hemiplegic stroke patients. Romanian Journal of Physical Therapy/Revista Romana de Kinetoterapie, 18(30), 56–63.

[B12] Hassan M. Terrien J. Marque C. Karlsson B (2011). Comparison between approximate entropy, correntropy and time reversibility: Application to uterine electromyogram signals. Medical Engineering & Physics, 33(8), 980–6. doi: 10.1016/j.medengphy.2011.03.010 21497127

[B13] Hu X. Wang Z. Ren X (2005). Classification of surface EMG signal with fractal dimension. Journal of Zhejiang University Sciences, 6B(8), 844–8. doi: 10.1631/jzus.2005.b0844 PMC1389869 16052721

[B14] Huang H. Wolf S. L. He J (2006). Recent developments in biofeedback for neuromotor rehabilitation. Journal of neuroengineering and rehabilitation, 3(1), 11. doi: 10.1186/1743-0003-3-11 16790060 PMC1550406

[B15] Kaufman M. Zurcher U. Sung P. S (2007). Entropy of electro-myography time series. Physica A: Statistical Mechanics and Its Applications, 386(2), 698–707. doi: 10.1016/j.physa.2007.07.045

[B16] Lei M. Wang Z. Feng Z (2001). Detecting nonlinearity of action surface EMG signal. Physics Letters A, 290(5–6), 297–303. doi: 10.1016/s0375-9601(01)00668-5

[B17] Merletti R. Parker P. A (2004). Electromyography: Physiology, engineering, and non-invasive applications. Hoboken, NJ: Wiley & Sons.

[B18] Nelson L. A (2007). The role of biofeedback in stroke rehabilitation: Past and future directions. Topics in Stroke Rehabilitation, 14(4), 59–66. doi: 10.1310/tsr1404-59 17698458

[B19] Padmanabhan P. Puthusserypady S (2004). Nonlinear analysis of EMG signals: A chaotic approach. Paper presented at The 26th Annual International Conference of the IEEE Engineering in Medicine and Biology Society San Francisco, United States, 1–5 September 2004. 10.1109/IEMBS.2004.1403231 17271750

[B20] Phinyomark A. Limsakul C. Phukpattaranont P (2011). Application of wavelet analysis in EMG feature extraction for pattern classification. Measurement Science Review, 11(2), 45–52. doi: 10.2478/v10048-011-0009-y

[B21] Phung D. Q. Tran D. Ma W. Nguyen P. Pham T (2014). Using shannon entropy as EEG signal feature for fast person identi cation. Paper presented at the European Symposium on Artificial Neural Networks, Computational Intelligence and Machine Learning Bruges, Belgium, 23–5 April 2014.

[B22] Wolf S. L (1983). Electromyographic biofeedback applications to stroke patients. Physical Therapy, 63(9), 1448–59. doi: 10.1093/ptj/63.9.1448 6351119

